# Assessing computer skills in Tanzanian medical students: an elective experience

**DOI:** 10.1186/1471-2458-4-37

**Published:** 2004-08-12

**Authors:** Miriam Samuel, John C Coombes, J Jaime Miranda, Rob Melvin, Eoin JW Young, Pejman Azarmina

**Affiliations:** 1 PRHO The Ipswich Hospital, Ipswich, Suffolk IP4 5PD, UK; 2 PRHO, Ealing Hospital, Middlesex UB1 3H, UK; 3 International Health Electives Co-ordinator, International Health and Medical Education Centre, University College London, London N19 5LW, UK; 4 PRHO, University College Hospital, London WC1E 6DB, UK; 5 Research Fellow, Centre for Health Informatics and Multiprofessional Education, University College London, London N19 5LW, UK

## Abstract

**Background:**

One estimate suggests that by 2010 more than 30% of a physician's time will be spent using information technology tools. The aim of this study is to assess the information and communication technologies (ICT) skills of medical students in Tanzania. We also report a pilot intervention of peer mentoring training in ICT by medical students from the UK tutoring students in Tanzania.

**Methods:**

Design: Cross sectional study and pilot intervention study. Participants: Fourth year medical students (n = 92) attending Muhimbili University College of Health Sciences, Dar es Salaam, Tanzania. Main outcome measures: Self-reported assessment of competence on ICT-related topics and ability to perform specific ICT tasks. Further information related to frequency of computer use (hours per week), years of computer use, reasons for use and access to computers. Skills at specific tasks were reassessed for 12 students following 4 to 6 hours of peer mentoring training.

**Results:**

The highest levels of competence in generic ICT areas were for email, Internet and file management. For other skills such as word processing most respondents reported low levels of competence. The abilities to perform specific ICT skills were low – less than 60% of the participants were able to perform the core specific skills assessed. A period of approximately 5 hours of peer mentoring training produced an approximate doubling of competence scores for these skills.

**Conclusion:**

Our study has found a low level of ability to use ICT facilities among medical students in a leading university in sub-Saharan Africa. A pilot scheme utilising UK elective students to tutor basic skills showed potential. Attention is required to develop interventions that can improve ICT skills, as well as computer access, in order to bridge the digital divide.

## Background

Developments in information and communication technology occur at an astonishing rate. The World Wide Web (WWW) doubled in size during the first 6 months of 2000 and by 2005 the number of Internet users is likely to pass the one billion mark [1]. This has had huge implications for medical practice throughout the world. One estimate suggests that by 2010 more than 30% of a physician's time will be spent using information technology tools [2]. But these developments are occurring in a world that many of our colleagues cannot access. The International Labour Organization's World Employment Report for 2001 noted that barely 6% of people in the world had ever logged onto the Internet, and 85–90% of these are in the industrialized countries [3]. In September 2000, the digital divide was highlighted by the World Health Organization as 'more dramatic than any other inequity in health or income' [4].

In a world afflicted by poverty, debt and HIV, why has the digital divide continued to trouble so many academics and development policymakers? The basic concern is that the spread of information and communication technologies in developed countries is leaving the rest of the world behind.

The development of online databases allows medical professionals throughout the developed world immediate access to hundreds of e-journals at the touch of a button, a striking contrast to the plight of many of their colleagues in developing countries who are forced to trawl empty libraries. Highlighting one of the greatest tragedies of the digital divide, it threatens the very communities that could benefit the most from the developments in ICT.

Many programmes have concentrated on increasing the number and spread of telephones and computers [5]. Other schemes have minimised cost barriers to accessing Internet resources [6]. Beyond this classic access gap, several other factors have been identified as contributing to the divide. These include a gap in ability to use ICT, measured as the skills base; a gap in the actual use, measured as amount of time spent utilising ICT facilities [5]; and a gap in the impact of use, measured by financial, economic and clinical returns. In other words, equipment alone is useless unless people are able to use it effectively and informed of the potential benefits of its use.

During January and February 2003 we studied ICT skills of medical students at Muhimbili University College of Health Sciences (MUCHS), University of Dar es Salaam, in Dar es Salaam, Tanzania. We also report a pilot intervention of peer mentoring training in ICT by medical students during the elective period.

## Methods

### Setting

MUCHS is the largest medical school in east Africa and the only public medical school in Tanzania. A lack of resources has resulted in the university library being filled with out-dated textbooks. The majority of its graduates go on to serve rural communities throughout Tanzania and its neighbouring countries, often as the only qualified doctor serving populations of over 100,000.

Access to computer facilities is a key problem. The University of Dar es Salaam is aiming for a ratio of one computer for every ten students. One-hundred-and-twenty computers would therefore be required for the 1200 MUCHS students. Help from international donors has allowed MUCHS to secure the presence of 40 computers, but only 25 are fully functional at any one time. Of these, less than half were connected to the Internet or loaded with basic software, bringing the real ratio of students to adequately equipped computers to around 100:1. Most MUCHS students found commercial Internet cafes too expensive to use on a regular basis. The cost of one hour at an Internet café can often be as high as $1, an important limiting factor considering that over 50% of an estimated 36 million people live in extreme poverty, surviving on less than US $1 per day [7].

### Methods

The abilities and attitudes of the fourth year MUCHS medical students (MD4s) towards ICT was assessed using Questionnaire 1 [see Additional File 1], an adapted version of a questionnaire developed by Jeannette Murphy j.murphy@chime.ucl.ac.uk at the Centre for Health Informatics and Multiprofessional Education (CHIME, ) in London, UK, to assess ICT skills amongst first year medical students (MD1s) attending University College London (UCL). The questionnaires were distributed to all MD4 students by Tanzanian student representatives, to be filled in independently, and were then collected by the representatives.

**Figure 1:**
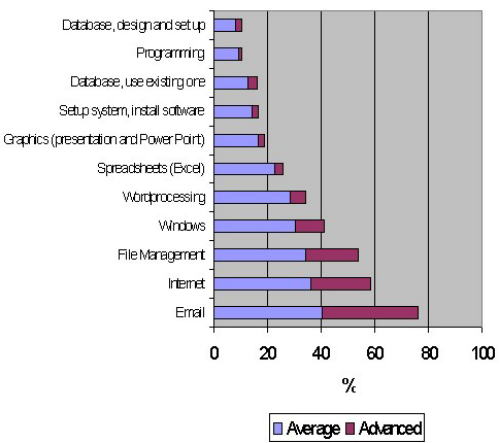
**Generic ICT Skills of 92 Medical Students at Muhimbili University College of Health Sciences. **The data are shown as percentages of students reporting average and advanced competences.

**Figure 2:**
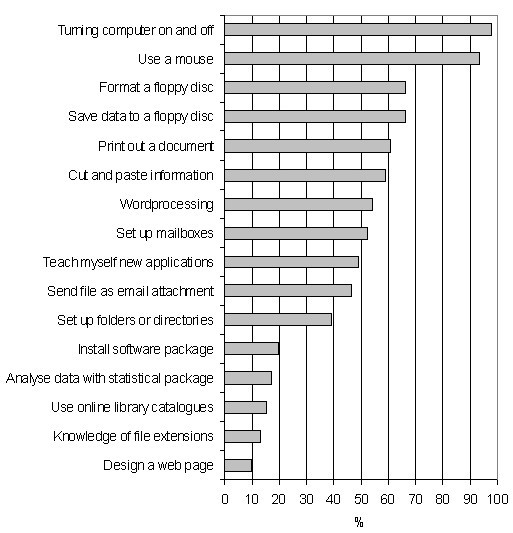
**Specific ICT Skills of 92 Medical Students at Muhimbili University College of Health Sciences. **The data are shown as percentages of students reporting to have the abilities to perform these tasks.

The questionnaire addressed different ICT-related variables associated with generic skills (Figure 1) and specific skills (Figure 2). A self-reporting assessment of competence (none, very basic, average or advanced, the equivalent of 0, 1, 2 or 3 points respectively) on several topics was evaluated as part of a generic ICT score. A specific ICT score aimed to address similar abilities (presence or not, 0 or 1 point) but asking about their abilities to perform such tasks. The generic ICT score has a range between 0 and 33 (11 items studied), and the specific one between 0 and 16 (16 items in total).

Information related to frequency of computer use (hours per week) and years of computer use was also gathered. Access to computers at home or at educational facilities, last time of computer use, reasons for use and resources for reference in students' medical studies was also evaluated.

The data were analysed using SPSS v.11 to calculate frequencies and percentages. Pearson Rho coefficient was used to look at the correlation between variables, with logarithmic transformation where necessary. Comparisons of frequencies before and after tutoring were performed using Wilcoxon's test. Data are shown as mean (± SD) for normally distributed data and median (interquartile [IQ] range) for skewed data.

## Results

Ninety two (72.7%) of 120 Tanzanian MD4s completed the questionnaires. 76% of them did not have a computer at home and 74% never use a computer as part of any course either at school or university. Only 48 students (52%) felt that they understood the basic terminology and concepts of computing.

The mean (± SD) of the generic and specific ICT scores were 11.1 (± 7.6) (out of a maximum score of 33) and 7.7(± 4.1) (out of a maximum score of 16) respectively. The two scores were significantly correlated, r = 0.81, p < 0.001.

The highest levels of competence, assessed using the generic ICT score, were for email, Internet and file management (see Figure 1). For the remaining items most respondents reported low levels of competence. Over 60% of the Tanzanian students in each of the generic areas indicated that they had taught themselves these skills. Of the remainder most students had learnt the skills at school, with a small number learning them at work.

The results for the tasks evaluated in the specific ICT score are shown in Figure 2. Of 16 skills evaluated, only 2 (12.5%) were present in 90% of the participants. The majority of skills were found in between 40 and 60% of participants.

The majority of the students claimed to use the available computers very regularly, 25% of students using them daily and nearly two-thirds at least once a week. The median hours per week of computer use was 3.8 (2–10). The median years of computer use was 3 (2–5) years.

The main reasons for using a computer during the last year was to communicate by email (75%), Internet navigation (33%), learning purposes (27%), and to prepare reports (22%). Only 21 students (23%) had ever consulted an electronic journal, and nearly 70% did not use any electronic resource. The participants unanimously agreed that medical students should receive training in the use of computers.

### Piloting a peer mentoring training scheme

During our time in Tanzania we piloted an ICT peer mentoring training scheme aimed at the fourth year Tanzanian medical students whose skills had been assessed. We utilized tutorials that had been developed by CHIME for underskilled UCL medical students [8]. Topics covered included file management, Internet, email, word processing, Excel and PowerPoint.

The willingness of the Tanzanian students to participate was identified as vital to the success of the scheme, since one of our main concerns was that we would we be seen as a neo-colonial force trying to impose our values. This was not the case: the response from the students was overwhelmingly positive. The majority of those who returned the questionnaires wished to receive peer mentoring training.

Four tutorials (1.0–1.5 hours) were conducted utilising MUCHS computers. The course covered computing skills such as file management, word processing, spreadsheet use, Internet access and email.

Competence levels were compared for 12 students before and after 4 to 6 hours of peer mentoring training using Questionnaire 2 [see Additional File 2]. The generic ICT score in these students increased from 8.8(+5.2) to 18.4 (+4.8) out of a maximum score of 33 (p = 0.007). The specific ICT score increased from 6.8 (+3.6) to 12.1(+2.2) out of a maximum score of 16 (p = 0.003). There were significant improvements (p < 0.05) in 7 of the 11 components of the generic ICT score and 5 of the 16 items of the specific score.

## Discussion

We have assessed ICT competence of a representative sample of 4th year medical students in a Tanzanian medical school and have found substantial limitations in computing skills. A mean generic score of 11.1 would be equivalent to approximately 1 point (very basic) in each of the 11 skills studied. The corresponding score among first year UK medical students in 2002 was 18.5. Initially at UCL, students with an overall score of less than 10 are considered to have low skills and are offered peer mentoring training. Using this criterion, around 50% of the Tanzanian students would fall into the low skills category compared with 9% of first year UCL medical students in 2002. Scores amongst UCL medical students have been rising in recent years (J Murphy, personal communication).

The mean of the specific ICT score was 7.7, in comparison with 11.9 for first year UCL medical students. (J Murphy, personal communication). The strong correlation between generic and specific scores suggests that the students were good at judging their own ability, thus decreasing a potential bias from self-assessment in our results.

Two studies from Nigeria show that there is poor knowledge of computer use. Ajuwon [9] reports that only 42.6% of the sample studied could use the computer. In Lagos [10], 79% of students had little or no computer skills. Although we did not evaluate self-reporting ability to use a computer, we can assume that this figure is similar considering the low averages of the scores reported in our study.

The Internet was the most common application utilised in our sample in Tanzania, especially for email communications (75%). This is concordant with the reports from Nigeria where 76.4% of first year clinical and nursing students in Ibadan [9] and 58% of final year medical and dental students in Lagos [10] have used email. This high rate of Internet and email use amongst medical students is also similar in other countries, such as Denmark [11], Finland [12], India [13], Malaysia [14] and the United Kingdom [15].

Around half of the Tanzanian students were able to print a document, cut and paste information from one application to another and word process an essay, letter or their CV. These results, clearly addressed through items in the specific ICT score, show us that even though the students are able to communicate by email and use the Internet, they cannot perform some basic skills necessary for working with files. The lack of confidence in use of packages such as PowerPoint and Excel was also noticeable in the reported score and in the reported reasons for using the computer.

The median duration of computer use was 3 years, meaning that most doctors in training had their first contact with a computer at university. Access is one of the main issues when one considers the fact that the current student/computer ratio at MUCHS is 100:1, compared to 35:1 in Portugal, 9:1 in the UK and 5:1 in Norway [16]. Seventy-six percent of students in our sample did not have a computer at home. This figure is in stark contrast with the 71.7% of first year medical students that do have access to a computer at home in Aarhus, Denmark [11] and 86% in California, USA [17].

These findings have substantial implications for addressing the digital divide in this population. These range from a marked limitation of knowledge of basic packages to the possibility of making incorrect assumptions that students who are able to communicate electronically are automatically able to perform basic tasks such as word processing. Graduates recruited by foreign institutions to pursue postgraduate training would face additional difficulties in their learning experience due to their problems with ICT. Also important is the fact that several academic institutions are now looking to expand their teaching programmes using online courses. Again, course organisers could erroneously enrol participants with a lack of basic ICT skills, based solely on the ability to communicate by email.

The peer mentoring training piloted in the present study shows promising results, achieving a doubling of competence scores. This programme [18,19], which uses foreign medical students visiting MUCHS to teach local ones, is a cheap intervention and feasible to replicate in the same context, and perhaps in other countries, in a similar way to the buddy system that has been suggested by Ajuwon in Nigeria [9].

## Conclusions

Our study has found a low level of ability to use ICT facilities among medical students in one university in sub-Saharan Africa. These findings reinforce the idea that more is needed to bridge the digital divide than simply increasing the number of computers. A pilot scheme utilizing visitors from the developed world to tutor basic skills showed potential, but it would be naïve to think that volunteers alone can bridge the gap. Some would argue that increasing the number and distribution of computers will eventually result in an improved skills base. Our experiences would support this. Tanzanian medical students are keen to learn new skills and are aware of the implications of being left behind in the technological revolution. The main concern of such a scenario is that without direct intervention the time required to attain these skills increases, to the further detriment of some of the world's most vulnerable societies.

## Competing interests

None declared.

## Authors' contributions

JCC, RM, MS, EJWY and JJM took part on the initial planning of the project. JCC, RM, MS and EJWY did the field work and the peer mentoring training to Tanzanian medical students. PA and JJM did the data input and analysis. MS, JJM and JCC wrote the initial version of the paper. All the authors contributed to subsequent versions of the paper.

## Pre-publication history

The pre-publication history for this paper can be accessed here:



## Supplementary Material

Additional File 1 **Questionnaire 1 **Instrument used to assess baseline ICT skills amongst MUCHS medical students. ©Jeannette Murphy Click here for file

Additional File 2 **Questionnaire 2 **Instrument used to assess skills after the ICT peer mentoring training amongst MUCHS medical students. © Jeannette Murphy Click here for file
