# Supplementary material for: Assessing computer skills in Tanzanian medical students: an elective experience
Source: BMC Public Health. 2004 Aug 12;4:37. doi: 10.1186/1471-2458-4-37 (PMC514556; doi:10.1186/1471-2458-4-37)
Supplement: Additional File 1 — Questionnaire 1 Instrument used to assess baseline ICT skills amongst MUCHS medical students. ©Jeannette Murphy [file 1471-2458-4-37-S1.RTF]

QUESTIONNAIRE for Medical Students 
Muhimbili University College of Health Sciences
Dar es Salaam, Tanzania

PLEASE COMPLETE AND RETURN TO GROUP REPRESENTATIVE

Computer Literacy Survey
CONFIDENTIAL
January 2003

We are five final year medical students from the UK, coming to Muhimbili University College of Health Sciences for our elective. We are planning a project about the use of computers in learning medicine in Tanzania and would appreciate your help. This questionnaire is designed to find out how much you currently use computers.

While at Muhimbili, we hope to show some of you how to gain more from computers, and how to use them more confidently. We are not experts, but would appreciate the opportunity to teach. As there are only five of us, we can only teach a few.

Please answer the questions. We will give another questionnaire to you later, to see if your use of computers has changed. Thank you. We're looking forward to meeting you.	

	
1.	Assessment of your IT Skills.  (Please complete the table below by ticking in the appropriate boxes)

Skills	Your Competence (tick ONE box for each skill)
(None, Very Basic, Average, Advanced)
	How skill acquired (tick ONE box - the main place skill learned)
(at school, at work, self-taught)	
	None	Basic	Average	Advanced	School	Work	Self	
word-processing								
Spreadsheets (Excel)								
graphics (presentation package)
(PowerPoint)								
email								
databases - use an existing database								
databases - design and set up a database								
Internet - World Wide Web								
Windows									
file management - save, delete, copy, merge, find								
programming								
set up a computer system, install software								


2.	Do you have any other Computing/IT qualification?	  YES     NO
		If YES, give name of course or qualification                                                                			

3.	When did you last use a computer? (Tick ONE box only)

I use one almost every day		
Within the last week		
Within the last month		
Within the last year		
More than a year ago		
Never used a computer		
4.	How long ago did you first start to use a computer? (please answer in either months or years) 
	________________ Months ago                                               Years ago


5.	Over the last year, how many hours per week did you use a computer? (in an average week)
__________________________ Hours per week


6.	List your main reasons for using a computer during the last year: 

(1)____________________________________________________________________

(2)____________________________________________________________________________

(3)____________________________________________________________________________


7.	Have you used a computer as part of a course at school, college or university?  

  YES      NO

8.	What resources do you use for reference in your medical studies? (Tick all that apply)

	Textbooks
	Photocopies of textbooks
	Lecture notes made by self
	Lectue notes made by others
	Videos
	Electronic textbooks
	Journals
	Online journals
	Others (please specify): ______________________________________________


9.	Do you feel that you understand the basic terminology and concepts of computing (e.g. words such as hardware, software, viruses, operating system, Windows environment, formatting discs)?

  YES       NO

10.	Have you ever used a computer-assisted learning package? (Please tick as appropriate)

		Yes, in high school
	Yes, at home
		I have never used a computer-assisted learning package
	Don't know. I have never heard this term before.
11.	How would you describe your typing skills? (Please tick ONE)

		I am completely unfamiliar with the basics of typing 
	I can type with two or three finger
	I am very competent but cannot touch type
	I can touch type

12.	Which of these statements best describes the way you feel about computers?

	I feel very confident using computers
	I feel I can cope
		I am completely lacking in confidence

13.	Please indicate which of these skills and knowledge you possess. (Tick all that apply)

	I know how to turn a computer on and off
	I am able to use a mouse
	I am able to format a floppy disc 
	I know how to save data to a floppy disc
	I can cut and paste information from one application to another
	I can print out a document
	I can set up folders or file directories
	I am able to wordprocess an essay or a letter or my CV
	I feel able to teach myself how to use a new application
	I am able to analyse data using a statistical package
	I am confident in using on-line library catalogues
	I can send a file as an email attachment
	I can set up mailboxes
	I am able to install a software package
	I understand different file formats (e.g. pdf, zip)
	I can design a web page


14.	Do you have a computer at home?   YES       NO


15.	Over the next 6 months, what computing/IT skills would you like to acquire (or improve)?

1)  …………………………………………………………..   2) ……………………………………

3) …………………………………………………………..


16.	Do you think medical students should receive training in the use of computers?  
	
  YES     NO


If YES, what type of training would you like to receive (if it were available)? 

		Timetabled small group teaching with trainer
		Self-study with study pack
			
17.	
What are your views on the relevance of computers to medicine?

	I expect that in my lifetime computers will deliver great benefits to doctors and 			their patients.
	I don't think that computers will ever play an important role in medicine.
	I am worried about the way in which computers are encroaching on medicine. 


Any comments you would like to make to help us to design the best learning opportunities for you and other medical students:
__________________________________________________________________________
____________________________________________________________________________________________________________________________________________________________________________________________________________________________________________________________________________________________________________


Age:            Sex:  Male   Female    

Your LAST Name (please print)                                        _____ _____________

Your FIRST Name (please print) ______________________________________


Your email address _______________________________________________

Your Home Address: 

____________________________________________________________________

____________________________________________________________________

____________________________________________________________________

(We do need your names so that we can set up our programme but the information you provide will not be retained; it will NOT go on your student records) 

Thank you for taking the time to complete this form. As we need time to analyse these forms, we'd be most grateful if you could return it to your group representative immediately. The questionnaire has been developed by the Centre for Health Informatics & Multiprofessional Education (CHIME), based at the Whittington Campus, on behalf of the Medical Undergraduate Teaching Unit of the Royal Free and University College Medical School. (If you have any queries about the questionnaire, please contact Jeannette Murphy, Senior Lecturer in Health Informatics)  (020 7288 5966)

Copyright of this questionnaire belongs to Ms Jeannette Murphy.
© Jeannette Murphy, Centre for Health Informatics & Multiprofessional Education, University College London, 2002
