# Supplementary material for: Assessing computer skills in Tanzanian medical students: an elective experience
Source: BMC Public Health. 2004 Aug 12;4:37. doi: 10.1186/1471-2458-4-37 (PMC514556; doi:10.1186/1471-2458-4-37)
Supplement: Additional File 2 — Questionnaire 2 Instrument used to assess skills after the ICT peer mentoring training amongst MUCHS medical students. © Jeannette Murphy [file 1471-2458-4-37-S2.RTF]

QUESTIONNAIRE FOR MEDICAL STUDENTS
Muhimbili University College of Health Sciences

IT SKILLS PROGRAMME
FEEDBACK FORM FOR PARTICIPANTS

CONFIDENTIAL
	

PLEASE COMPLETE AND RETURN AT THE END OF THE FINAL SESSION


COMPETENCE IN GENERAL IT SKILLS

1a	Thinking about your IT skills at this point in time, how would you rate each of your skills? (fill in Section 1 on your competence in each area)

1b	Which skills were covered by the computer tuition scheme? (fill in Section 2 of the table)

1c	Comparing your IT skills now to your skills before the computer tuition scheme, which have improved and which have stayed the same? (fill in Section 3 of the table below).


General IT Skills	Section 1
My competence now (tick one box for each skill)	Section 2
Was this skill covered in your training?	Section 3
My improvement for each skill (since the tuition began)	
	None	Basic	Average	Advanced	Yes/No	No change	Minor improvement	Major Improvement	
Word-processing (Word)									
email									
Windows									
Internet – World Wide Web									
File management -  save, delete, copy, find									
Databases – use an existing database									
Databases – design and set up a database									
Programming 									
set up a computer system; install software									
graphics & presentations (PowerPoint)									
spreadsheets (Excel)									


2	Do you feel that you understand the basic terminology and concepts of computing (e.g. words such as hardware, software, viruses, operating system, Windows environment, formatting discs)?
	 Yes			 No
3	From your personal perspective, did you benefit from the tuition sessions?

	 Yes   		 No


COMPETENCE IN SPECIFIC IT SKILLS

4a	Consider which of these IT skills you possess at this point in time (fill in section 1 of the table below)

4b	Which of these skills were covered on the tuition scheme? (fill in section 2 of the table below)

Skill	Section 1
Do you possess this skill? 
(tick one box)	Section 2
Was this skill covered during the tuition?
(tick one box)	
	Yes	No	Yes	No	
Switch a computer on and off 					
Use a mouse
					
Format a floppy disc
					
Save data onto a floppy disc					
Cut and paste data
					
Print documents
					
Set up folders or file directories					
Word process and essay or letter					
Teach myself how to use a new programme					
Analyse data using a statistical package					
Use internet library catalogues					
Send a file as an e-mail attachment 					
Set up a mailbox
					
Install a software package					
Understand different file formats.					


5	If you feel your IT skills have improved, how much did the tuition sessions you attended contribute to this improvement in your IT skills?

		 they were a major factor
		 they were a minor factor
		 they made no contribution


6	Which of these statements best describes the way you feel about computers?

	 I feel very confident
	 I feel I can cope
	 I am completely lacking in confidence

7	Has your confidence increased as a result of participating in this project?

	 Yes   		 No			
8	Do you think that peer tutoring is a good way of helping students such as you improve their IT skills?

	 Yes  			 No  			 Not sure

9	In your view, do you think the IT Skills Tuition should be implemented at MUCHS?

	 Yes  			 No  			 Not sure

10	On the whole, do you thinking the sessions achieved their main aim (helping students improve their IT skills)?

	 Yes   		 No

11	Was the course documentation useful? (handouts, booklet)

	 Yes   		 No


THE CONTENT AND ORGANISATION OF THE  TUITION. 

12	Do you think there were ...

	 too many sessions?
	 too few sessions?
	 about the right number of sessions?

13	Do you think there was the right mix of topics covered by the programme?

	 Yes  			 No


14	Any ideas for improving the IT training scheme?


	


15	Any additional comments:


YOUR NAME: 	Last Name ______________________________ First Name ___________________________
		E-mail address ______________________________________________________

Again, thank you for contributing to the programme and for filling in this form.

Thank you for taking the time to complete this form. As we need time to analyse these forms, we'd be most grateful if you could return it to your group representative immediately. The questionnaire has been developed by the Centre for Health Informatics & Multiprofessional Education (CHIME), based at the Whittington Campus, on behalf of the Medical Undergraduate Teaching Unit of the Royal Free and University College Medical School. (If you have any queries about the questionnaire, please contact Jeannette Murphy, Senior Lecturer in Health Informatics)  (020 7288 5966)

Copyright of this questionnaire belongs to Ms Jeannette Murphy.
© Jeannette Murphy, Centre for Health Informatics & Multiprofessional Education, University College London, 2002
